# Spatiotemporal analysis of psychoactive drug consumption in Brazil during the COVID-19 pandemic

**DOI:** 10.1371/journal.pone.0343552

**Published:** 2026-05-11

**Authors:** Evani Leite de Freitas, Rafael Santos Erbisti, Branca Grinberg-Weller, Daniel Claudiano Cabral Pinto, Elaine Silva Miranda

**Affiliations:** 1 Programa de Pós-graduação em Administração e Gestão da Assistência Farmacêutica, Faculdade de Farmácia, Universidade Federal Fluminense, Niterói, Brazil; 2 Departamento de Estatística, Instituto de Matemática e Estatística, Universidade Federal Fluminense, Niterói, Brazil; 3 Faculdade de Farmácia, Universidade Federal Fluminense, Niterói, Brazil; 4 Departamento de Farmácia e Administração Farmacêutica, Faculdade de Farmácia, Universidade Federal Fluminense, Niterói, Brazil; New York University - Abu Dhabi, UNITED ARAB EMIRATES

## Abstract

The COVID-19 pandemic had a significant impact on Brazil, although its effects were not homogeneous across the country. Socioeconomic disparities may influence epidemiological patterns during health emergencies, which impact population mental health. This study aimed to analyze the consumption of psychoactive medicines during the COVID-19 pandemic in Brazil and to characterize its spatiotemporal dynamics, identifying associated social, economic, and demographic factors. We used publicly available retail pharmacies dispensing data from January 2018 to September 2021 across Brazil’s municipalities. A statistical model was developed based on indicators of infrastructure, human capital, and labor and income. Generalized additive models were used to characterize the spatiotemporal distribution of drug consumption. In September 2021, average consumption in municipalities reporting COVID-19–related deaths was 22% higher than in municipalities with no reported deaths. Among the most frequently consumed medicines during the study period, second-generation antidepressants were predominant. Life expectancy, employment and education showed positive linear associations with consumption, whereas inadequate water supply and sanitation were inversely associated. Municipality income was positively associated with average consumption of psychoactive medicines, but those with a very high-income development index showed more complex dynamics. Our results suggest that COVID-19 mortality was associated with psychotropic medicines consumption and indicate the need to address socioeconomic vulnerability as a key component of public health emergency response. The COVID-19 pandemic had a significant impact on Brazil, although its effects were not homogeneous across the country. Socioeconomic disparities may influence epidemiological patterns during health emergencies, impacting population mental health. This study aimed to analyze the consumption of psychoactive medicines during the COVID-19 pandemic in Brazil and to characterize its spatiotemporal dynamics, identifying associated social, economic, and demographic factors. We used publicly available data from retail pharmacy dispensing from January 2018 to September 2021 across Brazilian municipalities. A statistical model was developed based on indicators of infrastructure, human capital, labor, and income. Generalized additive models were used to characterize the spatiotemporal distribution of drug consumption. In September 2021, average consumption in municipalities reporting COVID-19–related deaths was 22% higher than in municipalities with no reported deaths. Among the most frequently consumed medicines during the study period, second-generation antidepressants were predominant. Life expectancy, employment, and education showed positive linear associations with consumption, whereas inadequate water supply and sanitation were inversely associated. Municipality income was positively associated with the average consumption of psychoactive medicines, but those with a very high human development index for income showed more complex dynamics. Our results suggest that COVID-19 mortality was associated with psychotropic medicine consumption and indicate the need to address socioeconomic vulnerability as a key component of public health emergency responses.

## Introduction

The COVID-19 pandemic had a significant impact on Brazil, which ranks second globally in the number of deaths attributed to the disease, with more than 700,000 deaths having been officially recorded by the Brazilian Ministry of Health as of April 2025 [1,2]. The impact of the SARS-CoV-2 virus in the country may be linked to socioeconomic disparities, particularly those related to access to healthcare services and vulnerabilities in patient care, as well as political factors that emerged during the emergency period [1,3].

The influence of socioeconomic factors on the consequences of the pandemic across different social strata and countries may be summarized in the term “synergistic epidemic” or simply “syndemic”. This term refers to the interactions between diseases and social, environmental, or economic factors [4–6]. In a country marked by profound social inequality, such as Brazil, examining socioeconomic vulnerability is essential for understanding the broader public health challenges posed by the pandemic.

Disasters, health emergencies, and political and economic crises may have significant consequences for mental health. In such contexts, the use of psychoactive medicines, including antidepressants, anxiolytics, and hypnotics/sedatives, often increases [7–9]. During the COVID-19 pandemic, several studies reported a marked rise in the consumption of these medication classes, particularly antidepressants, anxiolytics, and hypnotics [10–13]. In Brazil, sales of psychoactive medicines, especially antidepressants, also increased during the pandemic period [14].

Analyses of data from 65 countries indicate that between 2008 and 2019, there was a 4.08% increase in the consumption of medicines related to mental health care. While the greatest absolute increase occurred in high-income countries, the highest relative growth (7.88%) was observed in upper-middle-income countries, such as Brazil [15].

Therefore, the aim of this study is to analyze the consumption of psychoactive medicines during the COVID-19 pandemic in Brazil and to characterize its spatiotemporal dynamics, identifying associated social, economic, and demographic factors, using publicly available dispensing data from retail pharmacies.

## Methods

### Study design

This is an ecological, descriptive study. According to guidelines issued by the Brazilian Health Regulatory Agency (Anvisa), all transactions involving controlled substances in private, retail pharmacies nationwide must be recorded, ensuring compliance with national regulations and enabling supply chain monitoring [16]. Since 2007, information on these transactions has been collected electronically, allowing monitoring at the national level. Currently, data collected by retail pharmacies are periodically submitted to the National System for the Management of Controlled Products (SNGPC) [17], an electronic platform and national database managed by Anvisa and made publicly available under the Federal Government’s Open Data Policy [18]. The SNGPC dispensing data, used as a proxy for consumption, represents the consumption dynamics within the private retail sector. The analysis covers a 45-month period, from January 2018 to September 2021, aiming to characterize consumption patterns before and during the pandemic across Brazil’s 5,565 municipalities, as per the 2010 Demographic Census. Data retrieval was performed in October 2022.

### Data sources

Medicine dispensing data at the municipal level were obtained from the SNGPC. Annual population estimates used in drug utilization equations were provided by the Brazilian Institute of Geography and Statistics (IBGE) for municipalities and Federative Units [19].

Epidemiological data on COVID-19 were retrieved from the Coronavirus Panel, a publicly available database maintained by the Brazilian Ministry of Health [2]. It includes information on confirmed cases and deaths, allowing for the analysis of associations between the consumption of psychoactive medicines and the epidemiological scenario. Data on cases and deaths were aggregated by month and municipality, covering the period from March 2020 to September 2021.

Data on urban infrastructure, human capital, work and income at the municipal level were obtained from the Atlas of Social Vulnerability, published by the Institute for Applied Economic Research (IPEA) [20]. These indicators are based on the 2010 Demographic Census, the most recent national census with available information. In addition to these census-based indicators, social vulnerability indices (SVI) provided by IPEA were also included. A total of 85 indicators covering infrastructure, income, poverty, education, and employment were considered. Although derived from 2010 data, these indicators capture structural characteristics that tend to remain relatively stable over time and were therefore considered suitable for characterizing the national territory. S1 Table shows the complete list of the indicators obtained.

### Selection of medicines

Medicines were classified according to the World Health Organization (WHO) Anatomical Therapeutic Chemical (ATC) Classification System and their corresponding Defined Daily Doses (DDD). The DDD, defined as the assumed average maintenance dose per day for a drug used for its main indication in adults, served as the unit of measure [21].

The selection process began by screening all active ingredients listed under the ATC/DDD methodology [21] within the pharmacological classes of antidepressants (N06A), anxiolytics (N05B), and hypnotics and sedatives (N05C). Ingredients without an assigned DDD were excluded.

From this initial pool of 36 active ingredients, we applied a second selection criterion: only those showing an increase in sales volume, in absolute numbers, between 2018 and 2021 were retained. This yielded a final list of 32 active ingredients, including clonazepam—a drug of particular concern in Brazil due to historical patterns of misuse [22]. As a final step, we selected only active ingredients that showed increased consumption across all regions during the period, resulting in 21 active ingredients: alprazolam, amitriptyline, agomelatine, bupropion, buspirone, clobazam, clonazepam, clomipramine, desvenlafaxine, diazepam, duloxetine, escitalopram, fluvoxamine, mirtazapine, nortriptyline, paroxetine, sertraline, trazodone, venlafaxine, vortioxetine and zolpidem.

This stepwise selection strategy was adopted to reduce heterogeneity and analytical noise introduced by medicines with stable or declining consumption, and to allow a more robust characterization of spatiotemporal patterns using the chosen statistical method and defined model.

### Data analysis

#### Construction of variables.

To enable spatial and temporal analysis, drug utilization figures are presented in DDD per 1,000 inhabitants per day [21]. The chosen indicator provides a population-based estimate of medicines use, relevant for the studied health context, and allows for comparisons between regions with different population sizes [21].

Equations (1) and (2) used to calculate the indicators of the number of defined daily doses (No. DDD) and DDD per 1,000 inhabitants per day are described below, according to Osorio-de-Castro (2000) [23]:


$$No.\;DDD=\;\frac{packages\;sold\;\times \;units\;per\;package\;\times \;active\;drug\;per\;unit\;(g)\;}{DDD}$$ (1)

$$DDD\;per\;\text{1},\text{0}\text{0}\text{0}\;inhabitants\;per\;day=\frac{No.\;DDD\;\times \text{1},\text{0}\text{0}\text{0}}{number\;of\;inhabitants\;x\;n^{\circ }of\;days\;in\;the\;analyzed\;period}$$ (2)

To analyze the association between the COVID-19 pandemic and the consumption of the selected medicines, two categorical variables were created to measure the monthly effect of reported deaths and cases in Brazilian municipalities. These variables represent an interaction between the presence of cases or deaths and the month within the pandemic period, enabling the identification of existing associations relative to periods without reported cases or deaths on drug consumption. Given that COVID-19 cases and deaths initially emerged in a few Brazilian municipalities at the end of March 2020, the indicator variables were defined starting in April 2020. The variables are described below:


$$\begin{array}{c} \overset{\text{(1)}}{\underset{i}{Z}}=\left\{ \;\;\;\;\;\begin{array}{c} \text{0},\;\;\text{if}\;\text{no}\;\text{cases}\;\text{in}\;\text{municipality}\;i\\ \text{1},\;\text{if}\;\text{case}\;\text{occurred}\;\text{in}\;\text{April}\;\text{2020}\;\text{in}\;\text{municipality}\;i\\ \begin{array}{c} \text{2},\;\text{if}\;\text{case}\;\text{occurred}\;\text{in}\;\text{May}\;\text{2020}\;\text{in}\;\text{municipality}\;i\\ \vdots \\ \text{18},\;\text{if}\;\text{case}\;\text{occurred}\;\text{in}\;\text{September}\;\text{2021}\;\text{in}\;\text{municipality}\;i \end{array} \end{array}\right. \end{array}$$

$$\begin{array}{c} \overset{\text{(2)}}{\underset{i}{Z}}=\left\{ \;\;\;\;\;\begin{array}{c} \text{0},\;\;\text{if}\;\text{no}\;\text{deaths}\;\text{in}\;\text{municipality}\;i\\ \text{1},\;\text{if}\;\text{death}\;\text{occurred}\;\text{in}\;\text{April}\;\text{2020}\;\text{in}\;\text{municipality}\;i\\ \begin{array}{c} \text{2},\;\text{if}\;\text{death}\;\text{occurred}\;\text{in}\;\text{May}\;\text{2020}\;\text{in}\;\text{municipality}\;i\\ \vdots \\ \text{18},\;\text{if}\;\text{death}\;\text{occurred}\;\text{in}\;\text{September}\;\text{2021}\;\text{in}\;\text{municipality}\;i \end{array} \end{array}\right. \end{array}$$

#### Selection of infrastructure, education and work and income indicators.

To select the infrastructure, education, and work and income indicators at the municipal level, a regression model was first fitted to the logarithm of the aggregate consumption of the 21 active ingredients over the entire analysis period (January 2018 to September 2021). Since the indicators used in the predictor do not vary over time, the objective was to assess their influence on the aggregate outcome for the entire period and initially identify non-significant coefficients. Therefore, the LASSO procedure was employed to select significant variables that statistically explain psychoactive medicines consumption variation [24]. Also, LASSO was chosen as the regularization technique because it induces sparsity in the coefficient vector, facilitating variable selection and interpretability [25]. The selected indicators are marked with an asterisk (“*”) in S1 Table.

#### Building a single, integrated database.

After selecting the socioeconomic indicators, a single, integrated database was created, harmonizing and standardizing the information in terms of time scale and geographic space. The complete database contains 250,425 rows, representing 45 months of data for each of the 5,565 municipalities. The columns in the database include the socioeconomic indicators selected through LASSO regression, as well as epidemiological indicators and information identifying the municipalities, time period, federation unit, and region. Information on indicators that do not vary over time, but only spatially, such as those obtained from the Atlas of Social Vulnerability, was replicated over time.

Information on drug dispensing was qualified and aggregated at the municipal and monthly levels for the analysis period. During the data qualification process, a small number of non-standard records with unusually high values were identified, which are likely associated with reporting errors rather than regular consumption patterns. To reduce the undue influence of these extreme values on model estimation, records exceeding the 95th percentile were excluded, that is, 5% of observations (amounting to 2,292,791 of 46,476,743 records), from the analysis for each psychoactive drug, considering all Brazilian municipalities over the 45 months of observation.

#### Statistical model description.

Different procedures were employed to adjust a statistical model for characterizing the consumption of the selected medicines across the 5,565 Brazilian municipalities. These procedures were based on indicators of infrastructure, human capital, and labor and income.

To characterize the spatiotemporal distribution of drug consumption, we used generalized additive models (GAM) [26], which allow for spatial and temporal smoothing as well as the inclusion of covariates. Cubic splines were adopted to model the nonlinear effects of continuous covariates, as well as temporal trends and seasonality and spatial nonlinearity, due to their ability to capture smooth and gradual relationships [27,28]. The GAM models relate the logarithm of psychoactive drug consumption in each municipality and month (from January 2018 to September 2021) to social, economic, and demographic indicators.

The model assumes that the variation in the logarithm of consumption, obtained after a log-transformation with the addition of a small constant (c = 0.01) to handle zero values, follows a normal distribution across municipalities and over time. The average consumption is modeled as a function of a predictor composed of the sum of the following components for the i-th municipality (i=1, …, 5,565), in month t (t=1,…, 45),


$${log(DDD}_{it}\;)\sim Normal\left(\mu_{it},\tau^{\text{2}}\right)$$ (3)

$$\mu_{it}=\beta_{\text{0}}+\;s(t)+s\left({long}_{i},{lat}_{i}\right)+\sum_{j=\text{1}}^{p}\beta_{j}X_{ji}+\sum_{l=\text{1}}^{q}s_{l}{(W}_{li})+\sum_{k=\text{1}}^{\text{1}\text{8}}\alpha_{k}Z_{ki}$$

where $\beta_{\text{0}}$ is the intercept; s(t) is a nonlinear smoothing term for the time index (corresponding to the chronological ordering of observations) used to estimate the time evolution curve of average consumption; $s\left({long}_{i},{lat}_{i}\right)$ is a bivariate smoothing term for the geographical coordinates (latitude and longitude) of the municipality centroids, used to capture spatial variation in average consumption; $X_{j.}$ represents the explanatory variables that vary only spatially, with linear effects and coefficients $\beta_{j}$, while $s_{l}{(W}_{l.})$ are smoothing functions capturing the nonlinear effects of a second set of explanatory variables $W_{l.}$. Additionally, $Z_{k.}$ represents a categorical variable that measures the association of the monthly reported deaths or cases in Brazilian municipalities and consumption of medicines. The function *s*() is a cubic spline.

A post hoc hierarchical partitioning analysis was performed to evaluate the independent contribution of the terms in the final GAM to the adjusted coefficient of determination. This analysis was conducted using the gam.hp R package, which allows the decomposition of the relative contribution of predictors in generalized additive models [29].

All GAM models were fitted using the gam() function from the mgcv package in R*®* [30]. The comparison and selection of the best model were based on the Akaike Information Criterion (AIC). LASSO regression models were fitted using the glmnet() function from the glmnet package in R*®*. Graphs and maps were generated using the ggplot2 package in the same software. Statistical significance was defined as p<0.05.

#### Ethics statement.

The data used in this study came from different sources all publicly available. All information was unidentified and aggregated. According to the exemption rule for non-identified aggregated public data in Brazilian ethical guidelines (Brazilian National Health Council Resolution N^o^. 510/2016) it is not necessary to submit for a Research Board for ethical waiver or approval.

## Results

Table 1 displays consumption data for the 21 selected active ingredients (indicated by *) dispensed from January 2018 to September 2021. To facilitate comparisons between the periods before and after the emergence of COVID-19 and to assess consumption trends over time, the 45 months of observation were categorized into three phases: pre-pandemic (January 2018 – February 2020), during the pandemic (March 2020 – September 2021), and the first year of the pandemic (March 2020 – March 2021). This classification allows for an assessment of how this variation occurred during the analysis period and the association with the pandemic context.

**Table 1:** Consumption of Psychoactive Medicines in Brazil before and during the COVID-19 pandemic period, in DDD per 1,000 inhabitants per day in selected periods, Brazil and Regions.

Active Ingredient	Pre-pandemic (jan/18 to feb/20)						During the pandemic (mar/20 to sep/21)						One year into pandemic period (mar/20 to mar/21)						Variation (%), jan/18 to sep/21)					
	BR	WC	NE	N	S	SE	BR	WC	NE	N	S	SE	BR	WC	NE	N	S	SE	BR	WC	NE	N	S	SE
ALPRAZOLAM*	1.61	1.66	1.17	0.51	2.24	1.89	1.83	1.96	1.44	0.62	2.43	2.11	1.80	1.92	1.40	0.60	2.40	2.08	13.9	17.8	22.7	21.6	8.4	11.6
AMITRIPTLINE*	0.34	0.41	0.32	0.19	0.50	0.31	0.39	0.47	0.39	0.23	0.59	0.35	0.39	0.47	0.39	0.22	0.61	0.35	17.1	14.4	22.8	23.9	17.7	12.9
AGOMELATINE*	0.02	0.03	0.01	0.01	0.04	0.03	0.03	0.04	0.01	0.01	0.05	0.03	0.03	0.04	0.01	0.01	0.05	0.03	22.1	20.0	29.8	18.2	20.9	21.9
BROMAZEPAM	0.33	0.28	0.28	0.11	0.51	0.35	0.32	0.28	0.28	0.12	0.48	0.33	0.32	0.28	0.28	0.12	0.49	0.34	−3.2	0.0	0.1	3.7	−6.0	−4.4
BUPROPION	3.04	3.81	1.02	0.90	7.34	3.19	3.76	4.68	1.31	1.14	8.59	4.08	3.63	4.52	1.22	1.06	8.40	3.94	23.6	22.9	28.0	25.9	17.1	27.8
BUSPIRONE*	0.03	0.02	0.01	0.01	0.08	0.03	0.05	0.04	0.02	0.01	0.13	0.05	0.04	0.04	0.02	0.01	0.12	0.04	72.2	100.5	135.1	95.5	60.1	67.3
CITALOPRAM	0.79	0.91	0.41	0.24	1.78	0.78	0.79	0.91	0.44	0.25	1.72	0.80	0.79	0.91	0.44	0.24	1.72	0.80	0.7	0.5	6.0	2.5	−3.9	2.3
CLOBAZAM*	0.15	0.24	0.11	0.06	0.23	0.15	0.17	0.26	0.13	0.08	0.25	0.17	0.17	0.26	0.13	0.08	0.25	0.17	14.2	8.7	23.0	30.6	10.8	12.1
CLONAZEPAM*	0.53	0.54	0.47	0.22	0.85	0.54	0.59	0.58	0.55	0.26	0.89	0.58	0.58	0.57	0.54	0.25	0.89	0.58	9.7	7.5	16.8	15.4	4.0	8.8
CLOMIPRAMINE*	0.62	0.75	0.34	0.24	1.24	0.64	0.67	0.83	0.39	0.26	1.33	0.70	0.67	0.82	0.38	0.25	1.32	0.69	9.1	10.6	12.9	9.8	6.9	8.9
DESVENLAFAXINE*	0.60	0.69	0.25	0.15	1.43	0.63	1.10	1.27	0.53	0.33	2.45	1.13	1.03	1.17	0.48	0.30	2.32	1.07	81.5	83.1	114.1	125.3	71.9	78.1
DIAZEPAM*	0.44	0.45	0.41	0.17	0.65	0.44	0.46	0.47	0.46	0.17	0.66	0.45	0.47	0.48	0.47	0.18	0.66	0.46	5.7	3.5	13.5	4.4	1.3	3.9
DULOXETINE*	0.55	0.67	0.27	0.19	1.25	0.55	0.70	0.84	0.37	0.24	1.52	0.71	0.68	0.82	0.35	0.23	1.49	0.69	28.0	24.8	38.9	27.4	21.3	30.6
ESTAZOLAM	0.02	0.01	0.01	0.00	0.02	0.02	0.01	0.01	0.01	0.00	0.01	0.01	0.01	0.01	0.00	0.00	0.01	0.01	−57.4	−47.4	−61.4	−66.6	−66.6	−52.6
ESCITALOPRAM*	1.96	2.23	1.10	0.62	4.41	1.91	2.70	3.12	1.76	0.95	5.74	2.55	2.60	3.01	1.67	0.89	5.59	2.47	37.5	40.1	59.6	52.3	30.2	33.4
FLUNITRAZEPAM	0.16	0.13	0.04	0.02	0.37	0.19	0.15	0.12	0.05	0.02	0.35	0.19	0.15	0.12	0.05	0.02	0.35	0.19	−2.2	−3.2	16.4	1.4	−6.5	−2.2
FLURAZEPAM	0.06	0.04	0.07	0.01	0.10	0.05	0.06	0.04	0.07	0.01	0.09	0.05	0.06	0.04	0.07	0.01	0.10	0.05	0.7	−2.9	3.4	12.2	−2.7	0.8
FLUVOXAMINE*	0.58	0.59	0.22	0.13	1.33	0.64	0.78	0.89	0.33	0.21	1.81	0.81	0.74	0.84	0.31	0.19	1.75	0.78	35.1	51.5	51.5	58.0	35.4	27.5
FLUOXETINE	0.90	1.13	0.67	0.39	1.69	0.85	1.00	1.22	0.80	0.50	1.67	0.97	0.99	1.20	0.78	0.48	1.69	0.97	10.9	7.6	18.0	27.8	−1.1	14.6
LORAZEPAM	0.27	0.20	0.17	0.05	0.47	0.33	0.26	0.19	0.16	0.05	0.43	0.32	0.26	0.19	0.16	0.05	0.44	0.32	−5.0	−5.1	−4.5	−5.5	−7.7	−3.7
MIDAZOLAM	0.06	0.04	0.03	0.02	0.11	0.06	0.06	0.04	0.03	0.02	0.11	0.06	0.06	0.04	0.03	0.02	0.11	0.06	−2.2	−7.2	−1.9	3.2	−2.0	−2.0
MIRTAZAPINE*	0.22	0.29	0.13	0.06	0.41	0.23	0.33	0.42	0.22	0.10	0.57	0.34	0.31	0.40	0.20	0.09	0.54	0.33	47.7	46.7	64.1	58.5	38.1	47.1
NITRAZEPAM	0.05	0.06	0.04	0.01	0.06	0.06	0.05	0.07	0.04	0.02	0.06	0.06	0.05	0.07	0.04	0.02	0.06	0.06	1.2	9.7	2.0	10.3	0.5	−0.7
NORTRIPTYLINE*	0.10	0.14	0.08	0.03	0.19	0.10	0.13	0.17	0.10	0.04	0.22	0.12	0.12	0.17	0.10	0.04	0.21	0.12	21.4	20.7	28.2	24.0	14.4	22.4
PAROXETINE*	0.78	0.83	0.45	0.29	1.70	0.77	0.91	1.01	0.59	0.38	1.90	0.88	0.90	0.98	0.57	0.37	1.87	0.87	17.9	21.6	31.0	32.2	11.9	15.5
SERTRALINE*	1.29	1.57	0.71	0.41	3.02	1.21	1.58	1.93	1.03	0.59	3.50	1.42	1.54	1.88	0.97	0.55	3.44	1.40	22.3	23.0	44.6	41.3	16.0	17.6
TIANEPTINE	0.00	0.00	0.00	0.00	0.00	0.00	0.00	0.00	0.00	0.00	0.00	0.00	0.00	0.00	0.00	0.00	0.00	0.00	−1.5	0.7	−19.9	58.4	2.5	−2.6
TRANYLCYPROMINE	0.01	0.00	0.00	0.00	0.01	0.01	0.01	0.00	0.00	0.00	0.01	0.01	0.01	0.00	0.00	0.00	0.01	0.01	5.0	61.2	−2.0	72.5	−9.6	9.3
TRAZODONE*	1.22	1.48	0.57	0.33	2.87	1.22	1.56	1.91	0.77	0.42	3.56	1.55	1.50	1.84	0.73	0.40	3.46	1.51	27.5	29.3	34.4	26.4	24.1	27.8
VENLAFAXINE*	6.40	6.28	2.88	1.57	15.28	6.69	8.13	8.17	4.04	2.01	18.91	8.38	7.82	7.80	3.84	1.85	18.27	8.09	27.0	30.0	40.5	28.2	23.8	25.3
VORTIOXETINE*	0.04	0.05	0.01	0.01	0.10	0.05	0.07	0.08	0.02	0.02	0.16	0.08	0.06	0.07	0.02	0.02	0.15	0.07	60.9	67.5	66.2	81.9	54.8	62.4
ZOLPIDEM*	0.85	1.14	0.41	0.27	1.89	0.84	1.33	1.79	0.77	0.51	2.64	1.33	1.27	1.72	0.71	0.47	2.57	1.28	56.8	57.0	86.0	90.7	39.6	58.4
ZOPICLONE	0.01	0.02	0.00	0.00	0.02	0.01	0.01	0.01	0.00	0.00	0.02	0.01	0.01	0.01	0.00	0.00	0.02	0.01	−3.7	−5.9	−9.9	37.3	4.0	−7.8

The symbol * indicates the active ingredients selected to the data analysis. BR = Brazil; WC = Central-West; NE = Northeast; N = North; S = South; SE = Southeast.

Before the pandemic, the consumption of venlafaxine (6.40 DDD/1,000 inhabitants/day) and bupropion (3.04 DDD/1,000 inhabitants/day) stood out at the national level, along with escitalopram, alprazolam, sertraline, and trazodone. Regional data analysis shows increased consumption in the South, where the use of medicines such as venlafaxine and sertraline exceed twice the national average.

As the pandemic progressed between March 2020 and September 2021, zolpidem consumption increased from 0.85 DDD/1,000 inhabitants/day in the pre-pandemic period to 1.33 DDD/1,000 inhabitants/day, with the largest contribution coming from the South region. A similar pattern was observed for desvenlafaxine, which consistently showed the highest consumption in the South, well above the national average across all analyzed periods. Desvenlafaxine also exhibited the greatest increase in national consumption between the pre- and post-pandemic periods, rising by 81.5% between January 2018 and September 2021.

**Fig 1:**
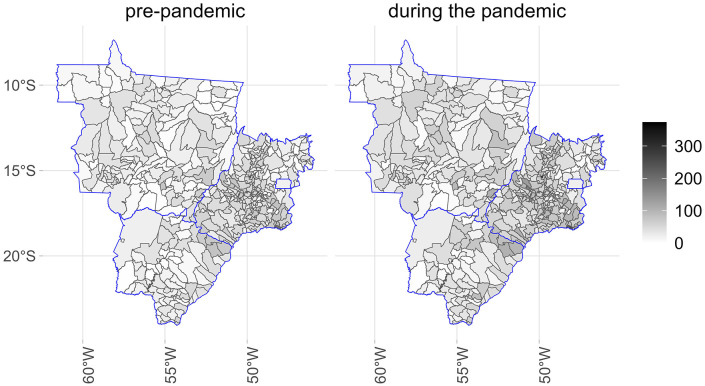
Municipal-level maps of medicine consumption (DDD/1,000 inhabitants/day) in the Central West Region before and during the COVID-19 pandemic (2018–2021). Source: Figure created by the authors. Municipal boundaries (shapefiles) were obtained from the Brazilian Institute of Geography and Statistics (IBGE) Open Data Portal: https://www.ibge.gov.br/geociencias/organizacao-do-territorio/malhas-territoriais/15774-malhas.html.

**Fig 2:**
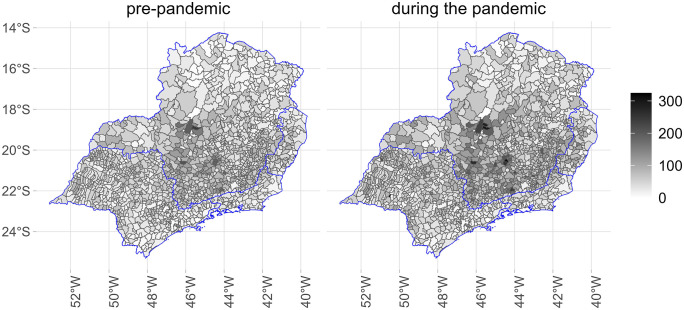
Municipal-level maps of medicine consumption (DDD/1,000 inhabitants/day) in the Southeast Region before and during the COVID-19 pandemic (2018–2021). Source: Figure created by the authors. Municipal boundaries (shapefiles) were obtained from the Brazilian Institute of Geography and Statistics (IBGE) Open Data Portal: https://www.ibge.gov.br/geociencias/organizacao-do-territorio/malhas-territoriais/15774-malhas.html.

**Fig 3:**
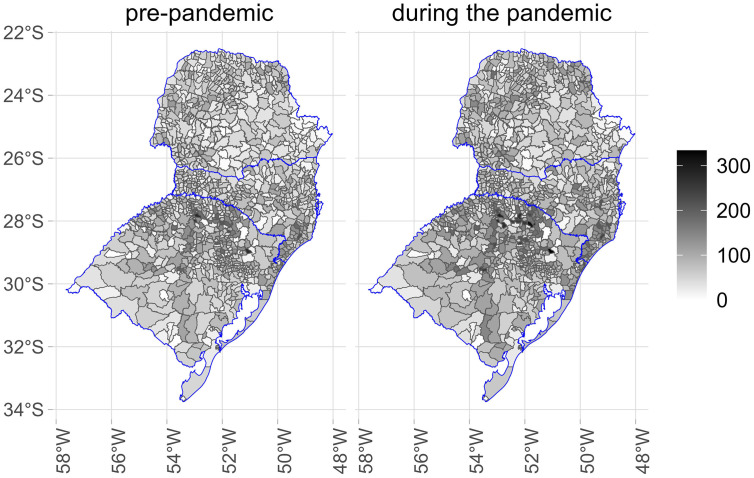
Municipal-level maps of medicine consumption (DDD/1,000 inhabitants/day) in the South Region before and during the COVID-19 pandemic (2018–2021). Source: Figure created by the authors. Municipal boundaries (shapefiles) were obtained from the Brazilian Institute of Geography and Statistics (IBGE) Open Data Portal: https://www.ibge.gov.br/geociencias/organizacao-do-territorio/malhas-territoriais/15774-malhas.html.

**Fig 4:**
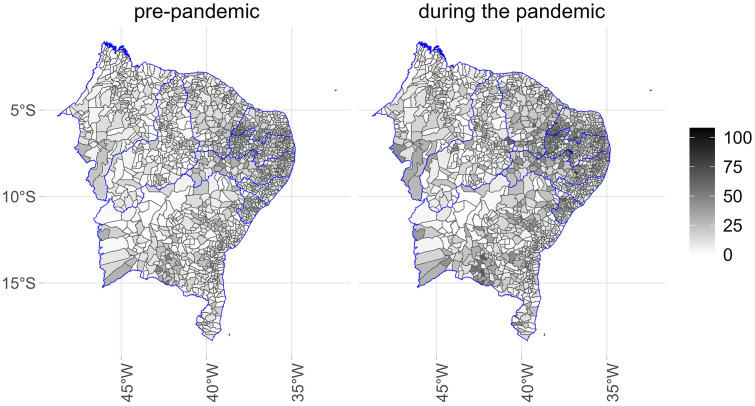
Municipal-level maps of medicine consumption (DDD/1,000 inhabitants/day) in the Northeast Region before and during the COVID-19 pandemic (2018–2021). Source: Figure created by the authors. Municipal boundaries (shapefiles) were obtained from the Brazilian Institute of Geography and Statistics (IBGE) Open Data Portal: https://www.ibge.gov.br/geociencias/organizacao-do-territorio/malhas-territoriais/15774-malhas.html.

**Fig 5:**
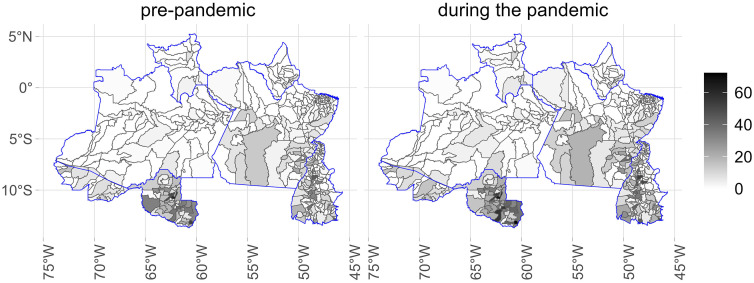
Municipal-level maps of medicine consumption (DDD/1,000 inhabitants/day) in the North Region before and during the COVID-19 pandemic (2018–2021). Source: Figure created by the authors. Municipal boundaries (shapefiles) were obtained from the Brazilian Institute of Geography and Statistics (IBGE) Open Data Portal: https://www.ibge.gov.br/geociencias/organizacao-do-territorio/malhas-territoriais/15774-malhas.html.

Figs 1–5 illustrate the consumption levels of medicines in each municipality across the five regions of the country. An increase in consumption during the pandemic was observed in all states. The North was the region with the lowest consumption, with high consumption foci concentrated in the states of Rondônia and Tocantins. In the Southeast, consumption was more evenly distributed, with municipalities in Minas Gerais standing out. In the South, consumption was uniformly spread across the territory.

### Final model and results

From an initial set of 85 socioeconomic, demographic, and infrastructure indicators, LASSO was used as a preliminary screening step to identify a reduced pool of candidate covariates [31]. The regularization parameter was selected via cross-validation, and the procedure resulted in 29 retained indicators (marked with “*” in S1 Table). These variables were not intended to be jointly included in the final spatiotemporal GAM, but rather to support the construction of parsimonious models. Several of the preselected indicators capture overlapping or highly correlated dimensions, making their simultaneous inclusion redundant. Therefore, multiple GAMs with different covariate combinations were fitted, and the final model was selected based on AIC comparisons. Although 29 indicators were preselected, only four remained statistically significant in the final model, representing those that best explained the spatiotemporal variation in drug consumption while maintaining model parsimony.

Initially, the GAM model described in Equation (3), in the section describing the statistical model, which define the spatiotemporal structure and the model’s additive predictor, was fitted with nonlinear smoothing over time and spatial coordinates to test the significance of $\overset{\left(\text{1}\right)}{\underset{i}{Z}}$and $\overset{\left(\text{2}\right)}{\underset{i}{Z}}$and to determine which of these variables should be included in the predictive structure of the model, that is, its additive predictor. Based on the AIC, it was decided to use the model that included the $\overset{\text{2}}{\underset{i}{Z}}$ variable. Next, models were fitted to test the previously selected SVI, after including the $\overset{\text{2}}{\underset{i}{Z}}$ variable, time and latitude and longitude via splines. Ten predictive structures were tested and using the AIC, the structure with the following indicators was selected: ind5 (percentage of people in households with inadequate water supply and sanitation), ind24 (Municipal Human Development Index – Income), ind25 (life expectancy at birth) and ind83 (percentage of employed individuals with complete higher education, aged 18 or older).

The best results were obtained when considering the non-linear association for the ind24 indicator. It should be noted that several indicators were highly correlated with each other, and the inclusion in the models tested considered only one of these indicators. Some indicators serve as proxies for socio-economic status (and perform better statistically than more direct economic indicators, which justifies their use in the model over others).

Table 2 presents the point estimates of the model’s linear coefficients, and increments of the analyzed outcome associated with each indicator. The data show that:

**Table 2:** Summary of the fit of the final model: estimate of coefficients, increments associated with indicators and p-values for significance tests.

Coefficients	Estimations	exp(Estimation)	Standard Error	p-value
**Intercept**	3.3449	–	0.1514	<2e-16
**Ind5**	−0.0029	0.9971	0.0004	1.29e-14
**ind25**	0.0243	1.0246	0.0021	<2e-16
**ind83**	0.0376	1.0384	0.0012	<2e-16

- for every 10 percentage points (p.p.) increase in the number of households with inadequate water supply and sanitation (*ind5*), an average reduction of exp(−0.0029×10)−1= −2.9% in the consumption of psychoactive drugs is expected, assuming all other conditions remain unchanged.
- For each one-year increase in life expectancy at birth (*ind25*), an average increase of exp(0.0243)−1=2.5% in the consumption of psychoactive drugs is expected, assuming all other conditions remain unchanged.
- For each p.p. increase in the proportion of employed individuals with a higher education degree (*ind83*), an approximate increase of exp(0.0376)−1=3.8% in medicine consumption is expected. Consequently, for every 5 p.p. increase in this indicator, psychoactive medication consumption is expected to rise by exp(0.0376×5)−1=20.7%, assuming all other conditions remain unchanged.

**Fig 6:**
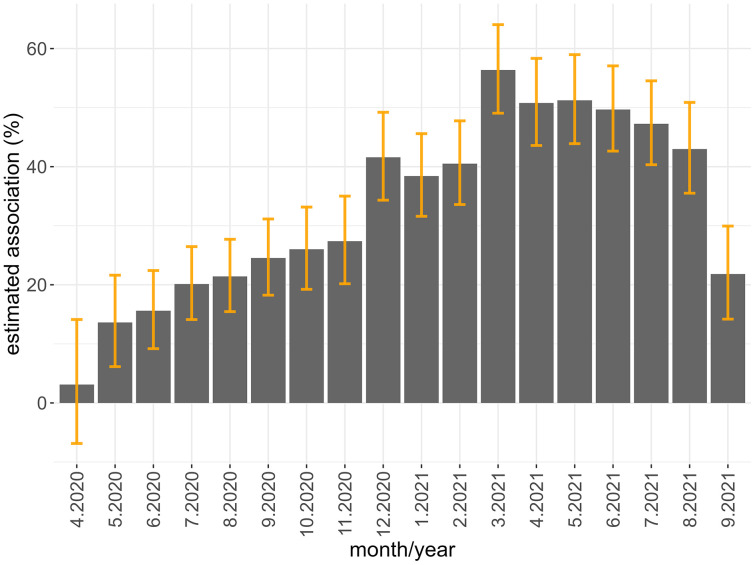
Estimated association between COVID-19-related deaths and psychoactive medicine consumption, with the respective 95% confidence interval, by month of the pandemic, compared to municipalities with no recorded deaths during the evaluation period. Source: Figure created by the authors using R software.

Fig 6 illustrates the estimated magnitude of association of variables indicating the occurrence of COVID-19-related deaths in municipalities each month from April 2020 to September 2021, compared to the pre-pandemic period (January 2018 to March 2020), on drug consumption. These results can be interpreted as a possible association of deaths during the pandemic with the consumption of psychoactive drugs.

From May 2020 until September 2021, all estimated coefficients were significant, following an upward trend until the beginning of the nationwide vaccination campaign in March 2021, when it reached an inflection point. From April 2021 onward, the magnitude of the observed association of COVID-19-related deaths on drug consumption began to decline compared to municipalities where no deaths were recorded during the analyzed period.

In September 2021, medication consumption in municipalities with reported deaths was, on average, 22% higher than in municipalities without deaths. This association remained stronger than that observed in May 2020.

**Fig 7:**
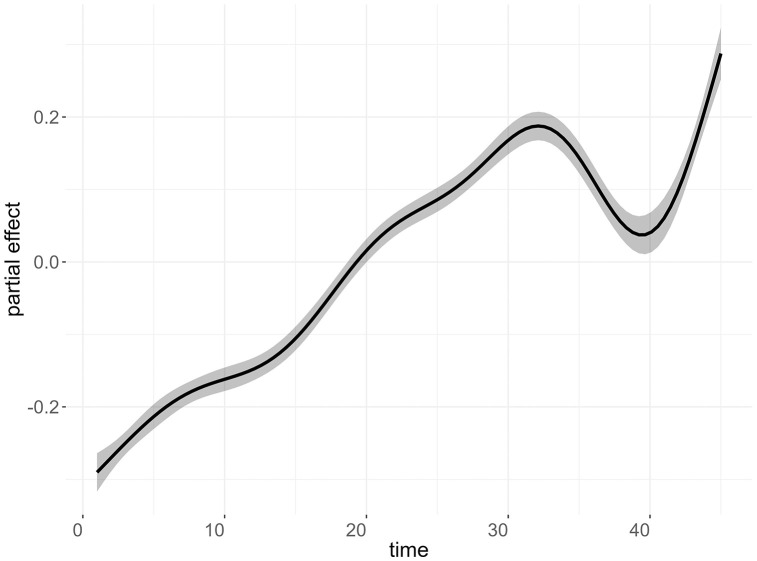
Smoothed partial temporal effects (on a logarithmic scale) on the consumption of psychoactive medicines in Brazil over the 45-month analysis period. The solid black line represents the point estimate of the temporal smooth, while the gray shaded band indicates the 95% confidence interval.

**Fig 8:**
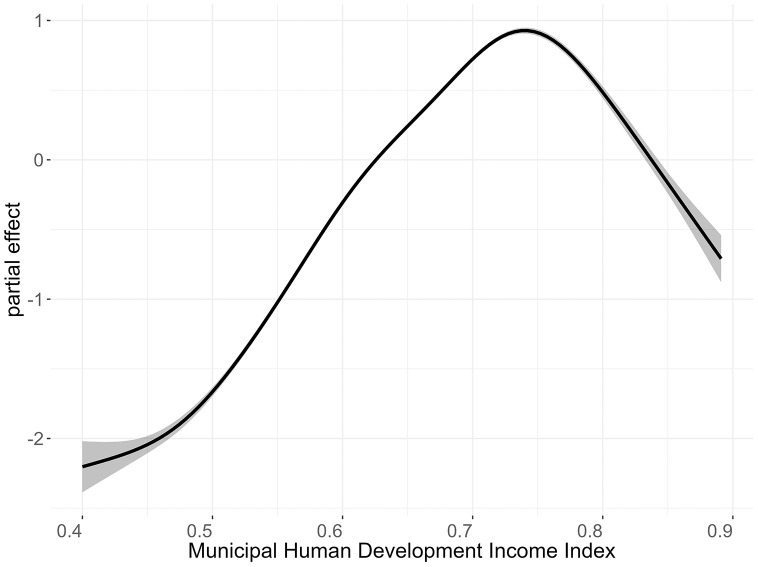
Smoothed partial effect of MHDI–Income (on a logarithmic scale) on the consumption of psychoactive medicines in Brazil. The solid black line represents the point estimate of the smooth effect, while the gray shaded band indicates the 95% confidence interval.

Figs 7 and 8 show, respectively, the smoothed curve of the time partial effect on psychoactive medicine consumption over the 45-month period, obtained from the final adjusted model, and the magnitude of variations in the Municipal Human Development Index – Income (MHDI-Income) on the consumption of the analyzed drugs.

It can be observed that municipalities with a low MHDI-Income exhibit a negative association with the average consumption, while populations with higher indices tend to have above-average consumption. Only ten municipalities have an MHDI-Income higher than 0.85, which may explain the lower-than-expected average consumption in municipalities with a very high-income development index. These municipalities are Nova Lima (MG), Vitória (ES), Niterói (RJ), Santana de Parnaíba (SP), Santos (SP), São Caetano do Sul (SP), Balneário Camboriú (SC), Florianópolis (SC), Porto Alegre (RS), and Brasília (DF).

The results of the post hoc analysis indicate that the spatial smooth term exhibits the largest independent contribution (35.7%), followed by the smooth term of ind24 – Municipal Human Development Index Income (25.9%) and two additional indicators, ind25 – Life expectancy at birth (14.64%) and ind5 – percentage of people in households with inadequate water supply and sewage disposal (13.9%). The remaining terms show smaller independent contributions, including ind83 – percentage of employed people with a university degree (6.2%), the temporal smooth term (1.9%), and the interaction term between COVID-19 deaths and months of the year (1.7%). These results are presented in S2 Fig, in Supplementary Material.

## Discussion

The methodological approach employed in this study, which integrates statistical modeling of dispensing data with the sociodemographic context, allows for a more profound qualification of consumption patterns. By going beyond descriptive analysis, this strategy enables the identification of critical points for intervention, providing evidence to support public policies aimed at reducing inequities in access to medicines.

Second-generation antidepressants were the predominant class of psychoactive medicines consumed in the Brazilian private market between 2018 and 2021, particularly sertraline and escitalopram (serotonin reuptake inhibitors), as well as venlafaxine and desvenlafaxine (selective serotonin and noradrenaline reuptake inhibitors). These drugs have a favorable safety profile, which justifies their widespread use, especially among those more susceptible to adverse effects, such as adults aged 60 and over [32].

Other pharmacological classes also showed notable consumption throughout the analyzed period, including benzodiazepines (e.g., alprazolam), bupropion (an atypical antidepressant widely used for smoking cessation), and trazodone (a second-generation antidepressant and serotonin receptor antagonist) [33]. Additionally, zolpidem, a sedative-hypnotic whose use has been spreading worldwide at a concerning rate [34], showed a notable increase in this study, with an almost 57% rise between January 2018 and September 2021.

From a regulatory perspective, increased consumption of psychoactive medicines during this period may be linked to changes in health legislation. In response to the pandemic, Anvisa introduced temporary regulations to facilitate access to medicines under special control during the health crisis. Specifically, it allowed for an increase in the quantity of medications dispensed per prescription and authorized their remote sale [35]. The private market played a critical role during the COVID-19 pandemic. The sector possesses high territorial capillarity across Brazil, serving as a primary access point for pharmacotherapy, and was of high importance when non-emergency public health services were often disrupted or avoided due to fear of infection [36]. Although access to psychoactive medicines was facilitated, changes in the regulatory framework compromised the oversight of these transactions. In December 2021, new legislation temporarily suspended the mandatory reporting of dispensing data by retail pharmacies to Anvisa through the SNGPC, causing a regulatory gap regarding controlled substances [37]. Ferreira et al., 2023, underpin the importance of the public availability of regulatory data regarding medicines to develop drug utilization research, especially in Brazil, where there are few sources of medicine dispensing data. Despite the vulnerabilities identified in the quality of registers, the magnitude of this public database allowed for a broad analysis during a critical period for the healthcare system [38,39].

Social, economic, and sociodemographic indicators are linked to healthcare access [40,41]. Our findings suggest a linear relationship between longevity, education and employment, and sanitation and the consumption of psychoactive drugs during the COVID-19 pandemic.

Almeida Andrade et al., 2021 [42] found an association between high SVI and risk of mortality in Northeast Brazil during the first year of the pandemic. The association of epidemiological indicators of COVID-19 and socioeconomic vulnerability has been described elsewhere, reinforcing the syndemic aspect of the health emergency [43,44]. In Brazil, it has also been described for other diseases that caused outbreaks or are endemic to the country, such as zika fever and tuberculosis [45,46].

Life expectancy at birth is one of the most widely used indicators of population health and reflects broader social and economic conditions, such as improved standards of living, and greater access to education and healthcare services [47,48]. Moreover, it is influenced by per capita health investment, availability and infrastructure of healthcare services, and broader social and economic factors, such as gross domestic product (GDP) and the proportion of economically active population [48].

In this study, the percentage of employed individuals with higher education was also associated with psychoactive drug consumption in the private market. The educational level of Brazilian workers has increased, which is directly linked to income levels, particularly in jobs that require higher qualifications [49].

The relationship between adequate sewage disposal, access to treated water, and health is well established and remains a pressing issue in Brazil. In 2019, diarrheal diseases, along with dengue, malaria, leptospirosis, and schistosomiasis, accounted for more than 273,000 hospitalizations, with higher incidence in areas lacking proper sanitation. The hospitalization rate for waterborne diseases was particularly elevated in the North and Northeast regions where sewage collection and treatment services are less comprehensive [50].

At the same time, individuals living in rural areas of the North and Northeast—predominantly black and without health insurance—reported limited access to healthcare services [51]. Consequently, the connection between adequate sewage disposal, income, and healthcare access, as documented in the literature, aligns with the findings of this study. Specifically, medicine consumption in the private market was lower among the most socioeconomically vulnerable populations.

Total health expenditure in Brazil has been increasing since 2006, with a growing share coming from household budgets. Data from the National Health Survey (Pesquisa Nacional de Saúde – PNS) indicate a rise in out-of-pocket spending on medicines between 2013 and 2019 [52]. Additionally, reliance on the National Health System (SUS) for obtaining medicines decreases as education and income levels rise [51,52]. This trend may be linked to the findings of the present study, which analyzed data from the private market. The observed association between higher income and improved indicators may reflect greater financial capacity for accessing pharmacological treatment.

In this study, Brazilian municipalities with higher per capita income presented higher consumption of the analyzed medicines. A cross-national analysis of data from 25 countries highlights persistent gaps in access to mental health treatment. Even in high-income countries, only 36.8% of respondents reported receiving treatment for mood, anxiety, or substance use disorders within a 12-month period, while in low-income countries, this figure dropped to just 13.7% [53].

Lima et al, 2008 found that per capita income was directly and independently associated with the use of medicines for common mental health disorders. Individuals of lower income reported experiencing mental disorders but did not report using medicines [54]. A similar pattern was observed in a cohort study of elderly individuals in another Brazilian city [55]. In this study, the highest consumption of psychotropic drugs was recorded in the southern region of Brazil. This region has a higher proportion of residents with income and lower inequality in the distribution of per capita household income [56].

Our findings contribute to building on the previously described association between socioeconomic development and higher psychotropic drug consumption, likely due to greater access to healthcare and the financial means to purchase medicines. However, the lower-than-expected average consumption in municipalities with very high MHDI-Income suggests a more complex dynamic.

In these more developed areas, there may be better access to non-pharmacological mental health treatments, such as therapy and counseling. In high-income areas, mental health treatment may emphasize lifestyle interventions (e.g., psychotherapy, exercise) rather than pharmaceutical measures [57]. This might differ from other areas, where individuals may rely more on general practitioners who prescribe medication as the first line of treatment. These cities might have lower social pressures, which could contribute to lower levels of mental distress [58].

This study aimed to explore possible associations between COVID-19 deaths and psychoactive drug consumption. The highest number of COVID-19-related deaths in Brazil was recorded in 2021, peaking between April 4th and 10th, with daily fatalities surpassing 3,000 individuals. Vaccination began in January 2021, and despite the peak in deaths in April, a trend of stabilization in the number of cases was reported from that point onward [59].

During this period, sixteen states and the Federal District had Intensive Care Unit (ICU) occupancy rates exceeding 90%, creating a highly critical scenario nationwide, which persisted throughout March and April. The high demand for intensive care beds not only limited access for the most severely ill patients but also impacted on the quality of care for those who received it [59]. These epidemiological data suggest that the rising trend in psychotropic drug use may be associated with heightened feelings of insecurity, anxiety, and uncertainty during this period.

Anxiety related to the virus and the perceived severity of the disease has been linked to poorer mental health outcomes [60]. In Brazil, the prevalence of individuals reporting anxiety and depression increased between May 2020 and April 2021, alongside a rise in the proportion of people expressing concerns about finances, food insecurity, and fear of the disease. Mrejen et al. identified a positive correlation between the rise in the daily death rate and reported mental health symptoms [61], a finding that aligns with the results of the present study. Additionally, Blanchflower & Bryson have identified that COVID cases were positively associated with worse mental health, i.e., symptoms such as anxiety, depression and worry. The magnitude of the declined as vaccination rates rose in 2021 and 2023 [62].

Time-associated fluctuations estimated in our model may be linked to aspects of the pandemic, as well as economic and political influences. In Brazil, deaths from intentional drug self-poisoning have been on the rise, particularly since 2016. Between 2003 and 2022, the suicide rate due to this cause increased by 264%, with psychotropic medicines being the most used pharmacological group [61,63]. Souza et al., 2024 [63] highlight a temporal correlation between this trend and the complex political and economic landscape that intensified in 2016, characterized by an economic recession, as well as the consequences of the COVID-19 pandemic.

This study has limitations. Our findings describe spatiotemporal patterns and associations between municipal socioeconomic profiles and psychoactive substance consumption, but they cannot establish a causal link between individual characteristics and medication use, given its ecological design, in which the units of analysis are municipalities rather than individuals. These results should be interpreted as indicators of collective health dynamics rather than individual clinical determinants.

Additionally, this study relies on dispensing data collected from private retail pharmacies, which do not capture actual medication use and do not represent total sales or consumption across the country during the study period. However, the growing importance of out-of-pocket health expenditure and the capillarity of private pharmacies in the country justify its use [36].

Another limitation is the use of socioeconomic indicators from the 2010 Demographic Census to analyze data from 2018–2021. This was necessary because the 2022 Census was delayed due to the COVID-19 pandemic and updated data were not available at the time of analysis and have not been released yet. We assume that socioeconomic factors typically undergo slow structural transformations dependent on long-term public policies.

It is important to interpret the spatiotemporal results hereby presented within the context of the SUS [64]. Brazil ensures universal and free access to healthcare and essential medications through national policies that remained structurally stable during the study period [65]. However, the management of the health system is decentralized to state and municipal levels. Consequently, while the legal right to access is uniform, the operational efficiency and specific strategies to promote drug access, especially during the health emergency, may have varied across different states and municipalities. Due to the national scale of this study, it was not possible to incorporate granular data on specific local policy adaptations. Thus, some of the observed spatiotemporal heterogeneity may reflect these unmeasured differences in regional and local healthcare management.

This study presents a comprehensive analysis of how epidemiological indicators might associate with medicines consumption, from a public health perspective, in one of the most affected countries in the world. The spatiotemporal analysis of psychoactive drug use in Brazil between 2018 and 2021 suggests that spatial location and structural social indicators, particularly education, account for most of the variation in consumption observed in our model in this study. These variations may have been shaped could be explained by public health measures such as vaccination, as well as social, economic, and political factors. Additionally, incorporating social vulnerability indicators contributes to identifying possible disparities in access to pharmacological treatment for mental disorders particularly in the context of health emergencies and restrictions on public resources.

## Supporting information

S1 Table Infrastructure, education, labour and income indicators calculated by IPEA based on the 2010 Demographic Census and selected indicators of medicine consumption. (DOCX)

S1 Fig Independent contribution (%) obtained via hierarchical partitioning of the adjusted coefficient of determination for the fitted GAM model. (TIFF)
