# Supplementary material for: Spatiotemporal analysis of psychoactive drug consumption in Brazil during the COVID-19 pandemic
Source: PLoS One. 2026 May 11;21(5):e0343552. doi: 10.1371/journal.pone.0343552 (PMC13160444; doi:10.1371/journal.pone.0343552)
Supplement: S1 Table — (DOCX) [file pone.0343552.s001.docx]

**S1 Table. Infrastructure, education, labour and income indicators calculated by IPEA based on the 2010 Demographic Census and selected indicators of medicine consumption.**

| **Cod** | **Indicator** | **Cod** | **Indicator** | **Cod** | **Indicator** | **Cod** | **Indicator** |
| --- | --- | --- | --- | --- | --- | --- | --- |
| ind1 | Social Vulnerability Index (SVI) | ind23 | Municipal Human Development Index (MHDI) - Education | ind45 | Population aged 1 to 3 | ind67* | Economically active population - 18 years and over |
| ind2* | SVI urban infrastructure | ind24* | Municipal Human Development Index (MHDI) - Income | ind46 | Population aged 4 | ind68* | % of population in households with electricity |
| ind3 | SVI human capital | ind25* | Life expectancy at birth | ind47 | Population aged 5 | ind69* | % of population in households with density > 2 |
| ind4 | SVI Income and Work | ind26 | Education sub-index – Educational Municipal Human Development | ind48 | Population aged 6 | ind70 | Illiteracy rate - 18 years and over |
| ind5* | % of people in households with inadequate water supply and sewage disposal | ind27 | % of people aged 18 or over with completed primary education | ind49 | Population aged 6 to 10 | ind71* | Illiteracy rate - 25 years and over |
| ind6* | % of the population living in urban households without sewage disposal | ind28 | School attendance sub-index - IDHM Educational | ind50 | Population aged 6 to 17 | ind72* | Per capita income of those vulnerable to poverty |
| ind7 | % of people living in households with a per capita income of less than half the minimum wage (from 2010) who spend more than an hour travelling to work | ind29* | % of people aged 5 to 6 years old at school | ind51 | Population aged 11 to 13 | ind73 | % of income from labour income |
| ind8 | Mortality up to 1 year old | ind30* | % of people aged 11 to 13 years old in the final years of primary school or having complete primary school | ind52 | Population aged 11 to 14 | ind74* | Gini Coefficient |
| ind9* | % of children aged 0 to 5 not attending school | ind31 | % of people aged 15 to 17 years old with complete primary education | ind53 | Population aged 12 to 14 | ind75 | % of employees with a formal contract - 18 years and over |
| ind10* | % of people aged 6 to 14 not attending school | ind32 | % of people aged 18 to 20 years with completed secondary education | ind54 | Population aged 15 or over | ind76* | % of employees without a formal contract - 18 years and over |
| ind11 | % of women aged 10 to 17 that had child | ind33 | Per capita income | ind55 | Population aged 15 to 17 | ind77* | % of public sector workers aged 18 and over |
| ind12 | % of mothers who are heads of household, have not completed primary school and have a child under 15 years of age | ind34 | Social Prosperity | ind56 | Population aged 15 to 24 | ind78 | % of self-employed - 18 years and over |
| ind13 | Illiteracy rate among the population aged 15 and over | ind35 | Total population | ind57 | Population aged 16 to 18 | ind79* | % of employers - 18 years and over |
| ind14* | % of children living in households where none of the residents has completed primary education | ind36 | Mortality up to 5 years of age | ind58 | Population aged 18 or over | ind80 | Degree of formalization of the employed - 18 years and over |
| ind15 | % of people aged 15 to 24 who do not study, do not work and have a per capita household income equal to or less than half the minimum wage (from 2010) | ind37* | Dependency ratio | ind59 | Population aged 18 to 20 | ind81* | % of the employed population with complete primary education - 18 years and over |
| ind16 | Percentage of people with per capita household income equal to or less than half the minimum wage (from 2010) | ind38* | Total fertility rate | ind60 | Population aged 18 to 24 | ind82 | % of employed with completed secondary education - 18 years and over |
| ind17* | Unemployment rate for the population aged 18 and over | ind39* | Ageing rate | ind61 | Population aged 19 to 21 | ind83* | % of employed people with a university degree - 18 years or older |
| ind18 | % of people aged 18 or over without complete primary education with informal employment | ind40 | Vulnerable population aged 15 to 24 | ind62 | Population aged 25 or over | ind84 | Average income of the employed population - 18 years and over |
| ind19 | % of people in households with per capita income of less than half the minimum wage (from 2010) and elderly dependents | ind41* | Women who are heads of household and have children under the age of 15 | ind63 | Population aged 65 or over | ind85 | % of employed with no income - 18 years and over |
| ind20 | Activity rate of people aged 10 to 14 | ind42* | Occupied population vulnerable to poverty who return from work every day | ind64 | Economically active population - 10 years and over |  |  |
| ind21 | Municipal Human Development Index (MHDI) | ind43* | Population in vulnerable and elderly households | ind65 | Economically active population - 10 to 14 years |  |  |
| ind22* | Municipal Human Development Index - Longevity | ind44 | Population under 1 year old | ind66 | Economically active population - 15 to 17 years |  |  |

* indicators selected by LASSO regression.
