# Supplementary figures and images for: Spatiotemporal analysis of psychoactive drug consumption in Brazil during the COVID-19 pandemic
Source: PLoS One. 2026 May 11;21(5):e0343552. doi: 10.1371/journal.pone.0343552 (PMC13160444; doi:10.1371/journal.pone.0343552)

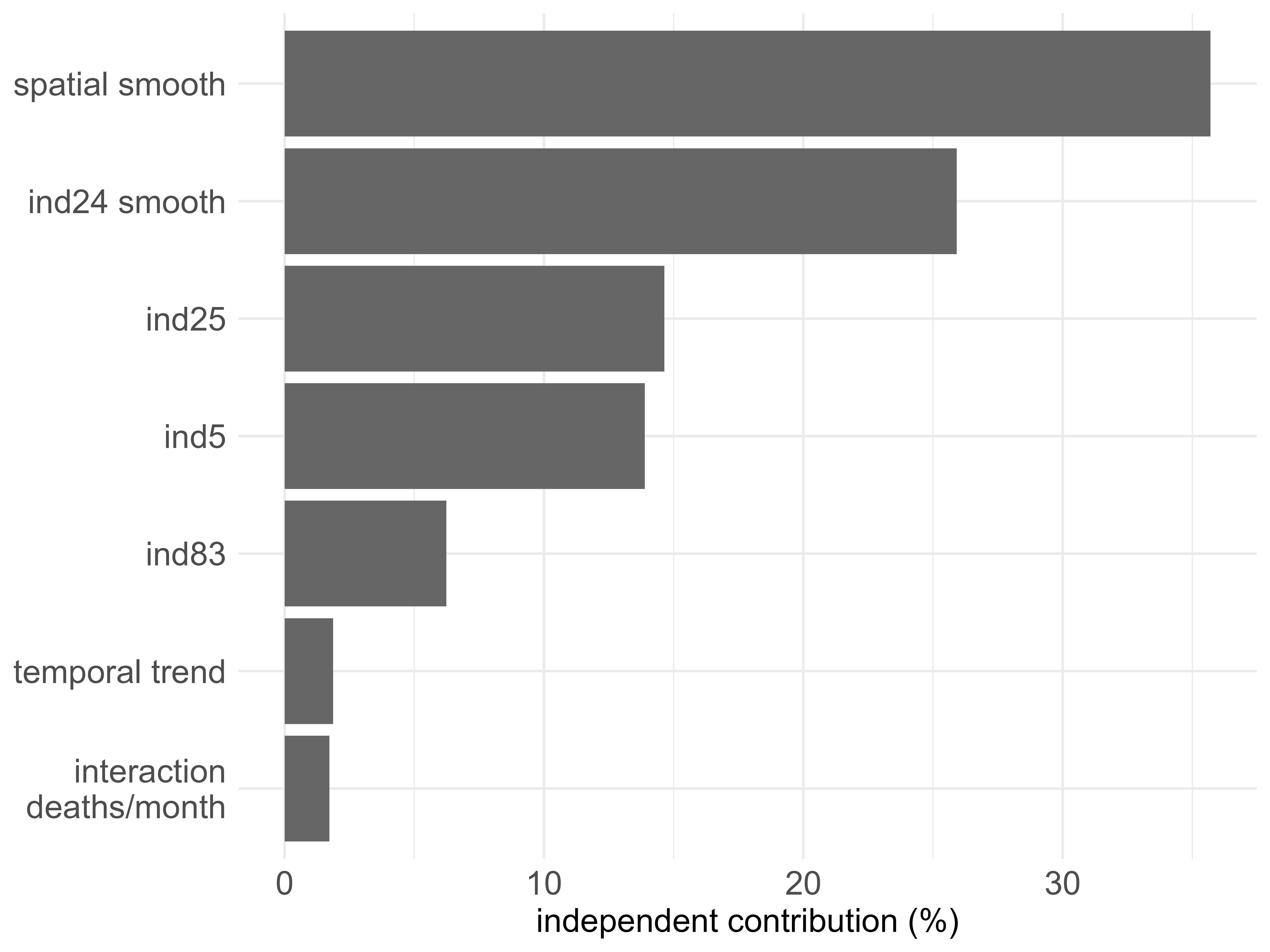

Supplement: S1 Fig — (TIFF) [file pone.0343552.s002.tiff]
